# Supplementary material for: ID1-Mediated BMP Signaling Pathway Potentiates Glucagon-Like Peptide-1 Secretion in Response to Nutrient Replenishment
Source: Int J Mol Sci. 2020 May 28;21(11):3824. doi: 10.3390/ijms21113824 (PMC7311998; doi:10.3390/ijms21113824)
Supplement: Supplementary file 1 [file ijms-21-03824-s001.pdf]

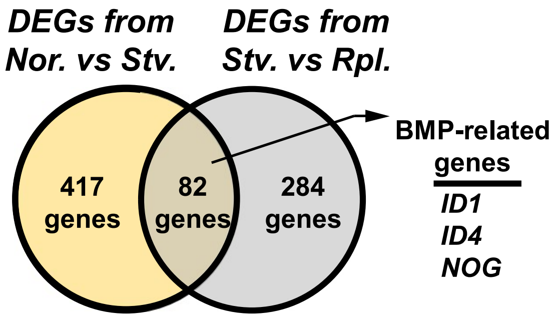

Figure S1. Count of overlapped genes in normal versus starvation and starvation versus replenishment DEGs. Total number of normal versus starvation DEGs is 499 genes. And total number of starvation versus replenishment DEGs is 366 genes. There are 82 overlapped genes between the two DEGs including ID1, ID4, and NOG.

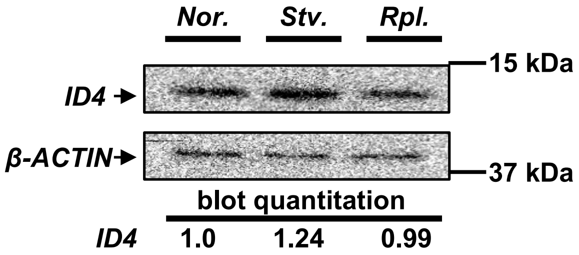

Figure S2. ID4 protein level according to nutrient status. Immunoblot of ID4 on normal, starvation and replenishment in GLUTag cells.

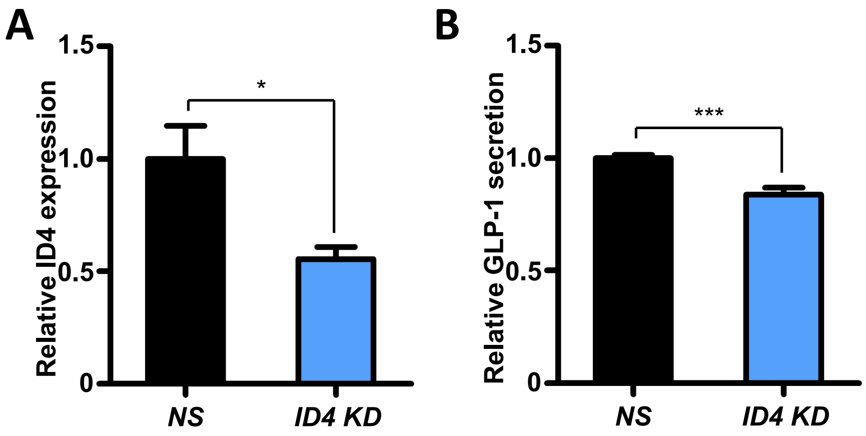

Figure S3. ID4-mediated BMP signaling modulates GLP-1 secretion in GLUTag cells. (A) mRNA expression level of ID4 gene with siRNA (n=3). **(B)** Basal GLP-1 secretion in GLUTag cells with ID4 siRNA (n=13). (NS; non-specific siRNA; KD; Knockdown with targeted siRNA)
